# 320-slice Computed Tomography Angiography Imaging Findings and Follow-up in A Patient with Aortic Coarctation Misdiagnosed as Hypertension: A Case Report and Literature Review

**DOI:** 10.7759/cureus.6529

**Published:** 2019-12-31

**Authors:** Peng Wang, Rui Jiang

**Affiliations:** 1 Department of Radiology, The General Hospital of Western Theater Command, Chengdu, CHN

**Keywords:** congenital anomalies, aortic coarctation, cta, aortic coarctation surgery

## Abstract

In this case, we report a simple coarctation of the aortic isthmus (CoA) in a patient without intracardiac abnormalities or patent ductus arteriosus, who was misdiagnosed as essential hypertension for over 20 years. The patient underwent X-rays, echocardiography, and 320-slice CT angiography (CTA). Among them, CTA comprehensively showed the diameter of the aortic coarctation, the anatomy and morphology of the heart and aortic arch, and the collateral circulation before surgery. It also accurately evaluated the postoperative status of the bypass vessel. This article highlights the excellent performance of 320-slice CTA in the diagnosis, surgical planning, and follow-up in CoA. Moreover, when adolescents suffer from refractory hypertension, the possibility of organic cardiovascular disease should be considered.

## Introduction

Coarctation of the aorta (CoA) is a congenital vascular malformation that is usually diagnosed in childhood but rare after adulthood, and usually involves intracardiac defects that require early surgical intervention [1-3]. Most untreated significant CoA patients die before the age of 30 years. Surgical timing and the approach to surgery depend on the location, length, and degree of stenosis, in addition to associated cardiovascular malformations. Surgical effects depend on a correct, comprehensive preoperative evaluation of these conditions, particularly when planning complex cardiovascular surgery [4,5]. This case has undergone detailed preoperative planning through 320-slice CT angiography (CTA) and achieved good surgical results.

## Case presentation

The patient was a 44-year-old male, who felt chest tightness and shortness of breath for more than 10 days. His symptoms were accompanied by dizziness, without chest pain. He was diagnosed with hypertension more than 20 years earlier and treated with antihypertensive drugs (no details of which were provided). His blood pressure (BP) was maintained at 180-160/100-80 mmHg. His physical examinations showed a body temperature: 36.9°C; BP​: upper limb 162/72 mmHg, lower limb 124/66 mmHg; body mass index: 23.4, without cyanosis or murmurs. Heart function of New York Heart Association class III and a heart rate of 60 beats per minute were also recorded.

**Figure 1:**
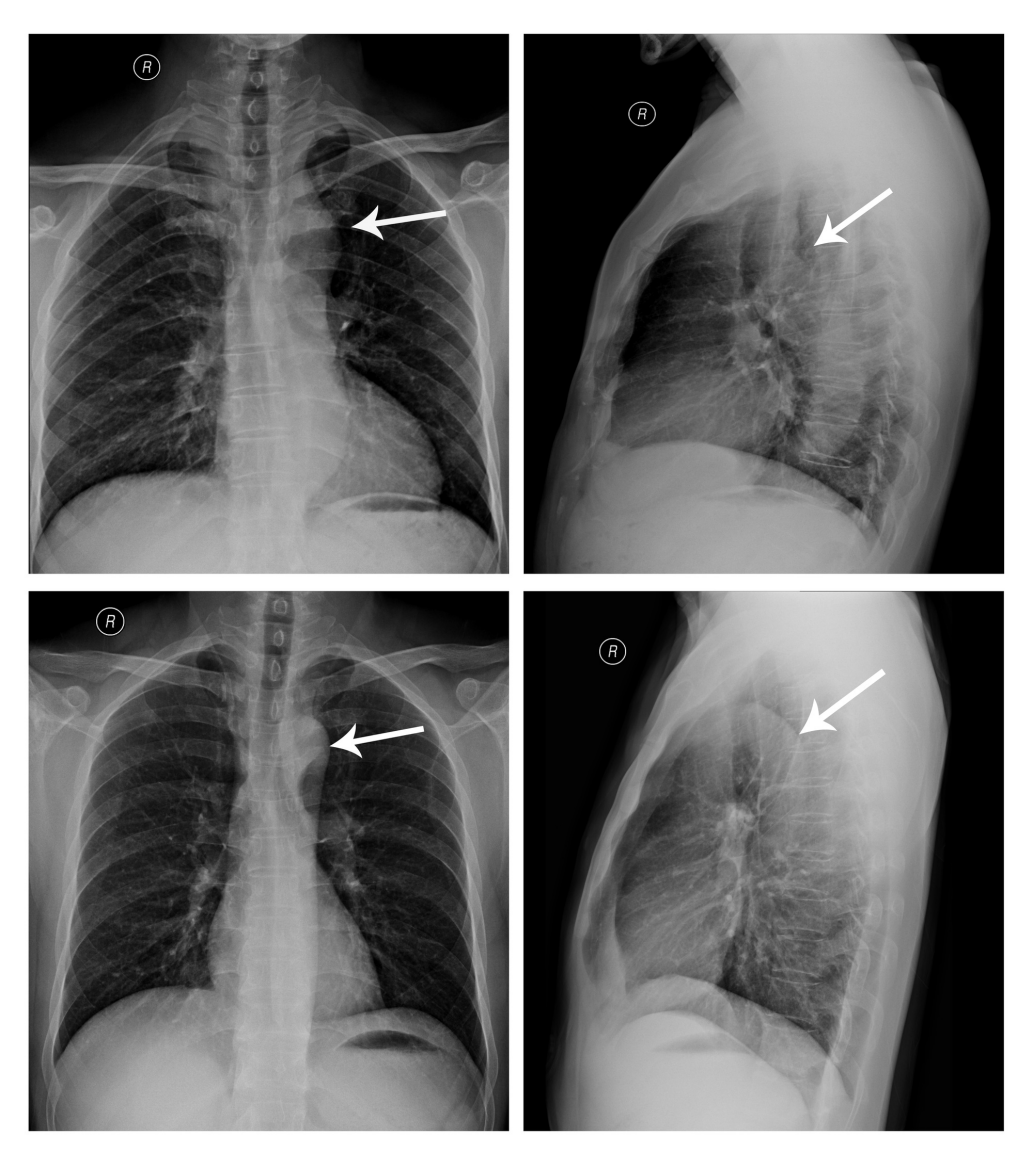
Chest radiograph image Chest radiographs of patients (upper rows) show that the aortic arch projection is incomplete (small white arrow) with contour deformity. A 44-year-old normal male chest radiograph is included for comparison and clearly shows an intact aortic arch (lower rows).

Chest radiographs showed a disappearance of the aortic knob and aortic isthmus discontinuity. A digital character “3” contour deformity was also observed (Figure 1).

**Figure 2:**
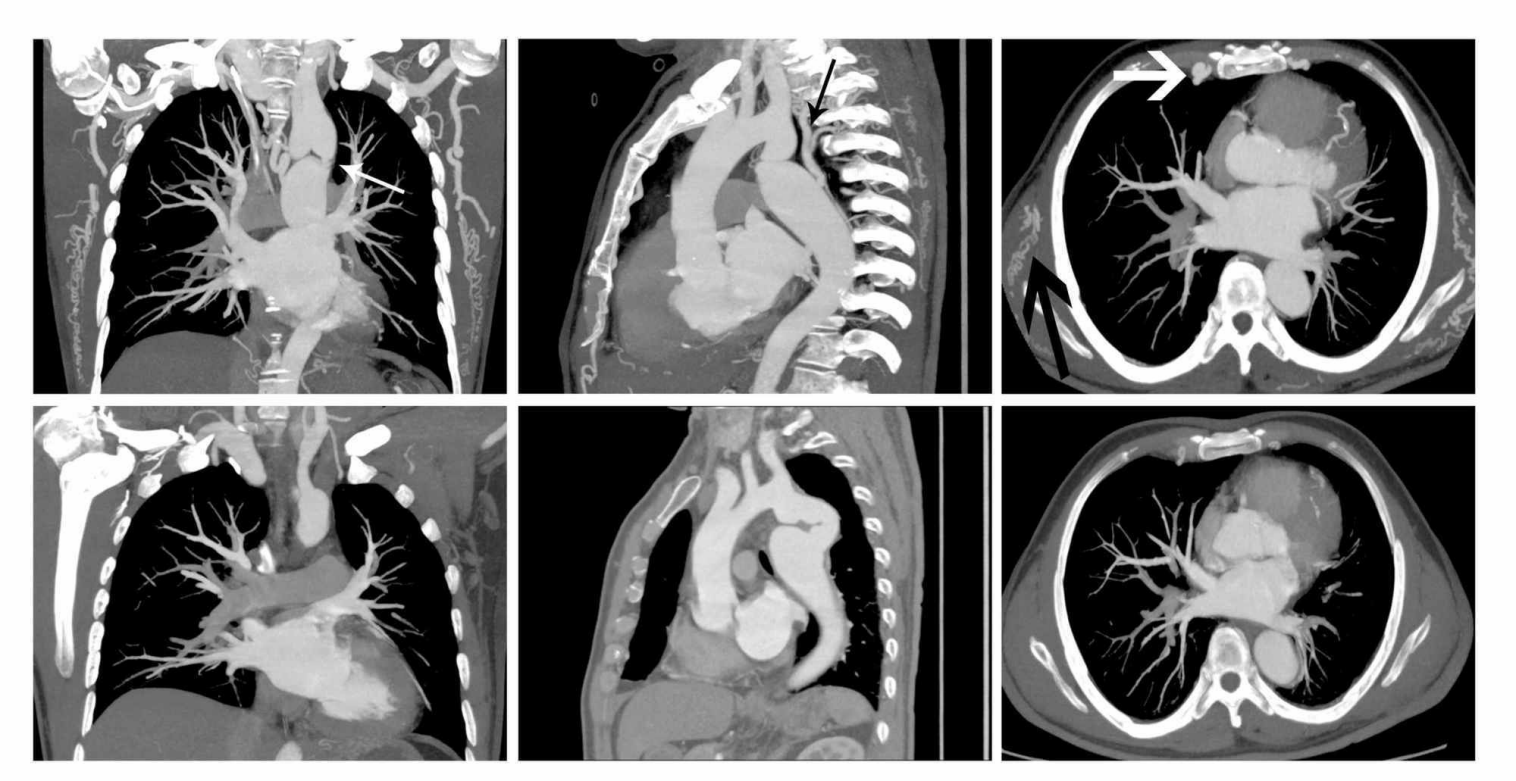
Vascular changes before and after surgery Stenosis of the CoA patient (small white arrow). The collateral vessels connect the descending aorta to the left subclavian artery (small black arrow). Distorted internal mammary arteries (thick white arrow), and extensive collateral vessels throughout the thorax (thick black arrow) before surgery (the upper row images). Bypass vessels were well structured and the enlarged collateral vessels shrunk after surgery (lower row images). CoA, coarctation of the aorta.

CTA examinations revealed aortic arch dysplasia. The aortic arch isthmus narrowed ~0.3 cm in diameter. The diameters of the upper and lower ends of the isthmus were 2.1 and 3.7 cm, respectively. Secondary aneurysm-like changes were observed. The blood supply was connected through collateral vessels between the two ends. No cardiac abnormalities in the heart were observed (Figure 2).

Echocardiography (ECG) showed mild regurgitation at the aortic valve, the mitral valve, and the tricuspid valve. The interventricular septum was thickened, and the atrial were enlarged. Left ventricular diastolic function became impaired, and ECGs revealed left ventricular hypertrophy.

The patient eventually underwent surgery for the resection of coarctation segment and interposition artificial vascular graft vs end-to-end to relieve the aortic arch constriction. Cardiac surgeons confirmed the presence of stenotic vessels in the aortic arch isthmus, measuring ~0.8 cm in length and ~0.3 cm in width. They connected both ends with artificial blood bypass vessel that was 2.0 cm in diameter. The pressure differences between the stenosis ends measured ~100 mmHg preoperative. After the surgery, the pressure decreased to 56 mmHg. His BP dropped to ​​130/74 mmHg.

**Figure 3:**
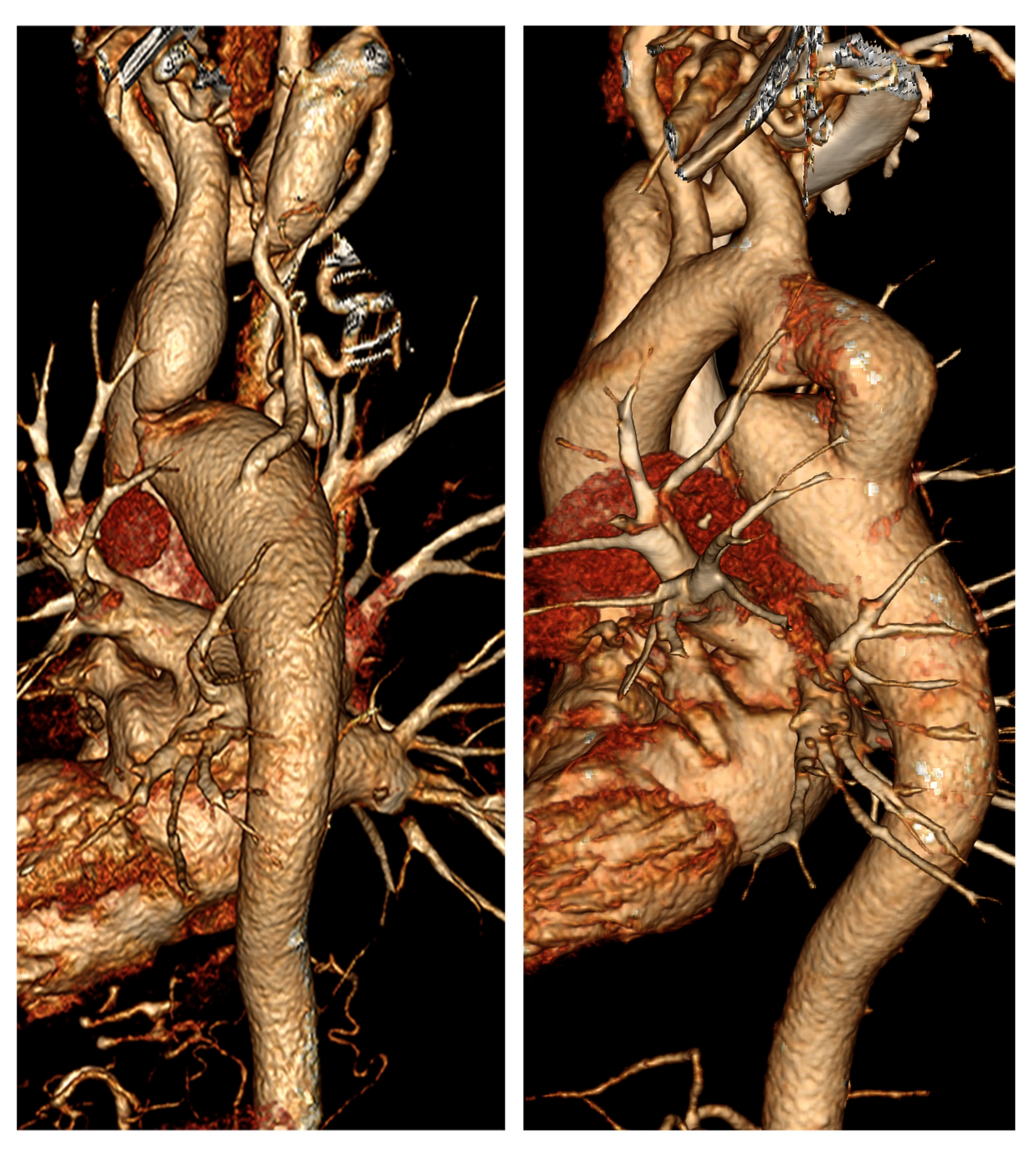
VR images VR images clearly show the location of CoA stenosis, the surrounding anatomy, and the performance of the bypass vessels after surgery. CoA, coarctation of the aorta; VR, volume reconstruction.

The patient was followed up with CTA three months after surgery. The bypass vessel (~1.6 cm in diameter) was well displayed, with no pseudoaneurysm formation and no leakage. The isthmus end (~1.2 cm in diameter) and descending aorta end (~2.8 cm in diameter) became smaller postoperatively. The number and diameter of collateral vessels also reduced (Figure 3).

## Discussion

In this case, an adult patient with simple CoA was misdiagnosed as hypertension over 20 years. Medical treatment was ineffective, and the patient suffered from grade 2 hypertension. CoA is a notable cause of unexplained hypertension in adults [6].

Routine chest radiographs identified abnormal aortic arch that failed to diagnose the type, position, and incidental conditions of the lesion. CTA (320-slice) was advantageous and revealed complex cardiovascular anatomical structures. With an improved detector width, images of the heart and large blood vessels can be obtained simultaneously through one-step examination, and optimized image reconstruction of the cardiovascular can be obtained [7-9].

Multiplanar reformation images showed no abnormalities in the heart chamber, interatrial septum, and the outflow tract. Maximum intensity projections (MIP) and volume reconstruction (VR) clearly showed aortic arch isthmus stenosis with thinning of the lumen. The case was therefore diagnosed as simple CoA.

Despite this seemingly simple anatomical performance, CoA is a complex medical problem with several associated anatomical and physiological abnormalities. The clinical treatment of CoA in adults usually involves multidisciplinary teamwork [10,11]. Probably due to his narrow lumen, our patient had time to establish an extensive collateral circulation in the early stages. MIP and VR clearly showed a thickly distorted collateral circulation network amongst the descending aorta, intercostal artery, internal mammary artery, and left subclavian artery. This vascular network was decisive for maintaining patient life.

The patients’ blood flow was reconstructed through the artificial blood vessel bypass. The arterial pressure differences between the two ends and general BP immediately decreased. Postoperative CTA showed the presence of no aneurysms in the artificial blood vessels. The aneurysmal dilatation at both ends of the original stenosis was smaller than previously, and the lateral branch vessels were significantly thinner.

Echocardiography and magnetic resonance imaging (MRI) failed to provide additional anatomical information in simple CoA compared to CTA. Echocardiography and MRI presented both the valve conditions and myocardial motion of each chamber wall in real-time [12,13]. Both remain necessary as supplementary methods.

## Conclusions

When adolescents suffer from refractory hypertension, the possibility of organic cardiovascular disease should be considered. In such cases, early imaging examinations are beneficial to the patient.

CTA reconstructs the anatomical images and permits the visualization of intracardiac malformations, the interrupted location of CoA, the collateral circulation, and measurements of relevant arteries that can guide surgical planning. CTA is therefore an effective means for CoA diagnosis, surgical planning, and postoperative evaluation.
